# Clinical characteristics of temporomandibular joint involvement in children with juvenile idiopathic arthritis

**DOI:** 10.1007/s40368-026-01183-1

**Published:** 2026-02-17

**Authors:** M. Kaarto, J. Snäll, M. Möttönen, A. Suominen, J. Hirvonen, H. Thorén

**Affiliations:** 1 University of Turku, Turku, Finland; 2 University of Helsinki, Helsinki, Finland; 3 Helsinki University Hospital, Helsinki, Finland; 4 Turku University Hospital, Turku, Finland; 5 University of Turku, Turku, Finland; 6 Turku University Hospital, Turku, Finland

**Keywords:** Juvenile idiopathic arthritis, Temporomandibular joint, Oligoarthritis, Seronegative polyarthritis

## Abstract

**Purpose:**

To describe and compare clinical characteristics of TMJ involvement in a sample of children with oligoarthritis and seronegative polyarthritis, and to identify factors increasing the odds for different types of TMJ involvements.

**Methods:**

A retrospective cross-sectional study of 62 children with oligoarthritis or seronegative polyarthritis and associated clinical TMJ involvement at the age of 15 years or younger (range 2–15, mean 7.9, SD 3.6, median 6.7).

**Results:**

Clinical TMJ involvement was frequently (35 children, 56.5%) diagnosed early (0 to 12 months) after JIA diagnosis. In five children (8.1%), TMJ involvement was identified prior to diagnosis of JIA. Subjective symptoms were far more infrequently reported (30 children, 48.4%) than abnormal clinical findings (57 children, 91.9%). The most common symptom was pain (28 children, 45.2%) and the most common clinical finding was disturbance of mandibular movements (46 children, 74.2%). Mandibular asymmetry was observed in 18 (29.0%) children. No significant differences in TMJ symptoms and clinical findings were observed between children with oligoarthritis and seronegative polyarthritis. MRI findings were absent in 20 (32.3%) of 62 children.

**Conclusions:**

No significant clinical differences between the two JIA groups were observed, likely due to the small sample size. However, important overall characteristics were observed: TMJ involvement frequently presented without any subjective symptoms, and one out of three children had mandibular asymmetry already at the time of diagnosis of TMJ involvement. The findings emphasise the importance of regular examinations of the TMJ in children with JIA in order to prevent facial growth disturbance and malocclusion.

## Introduction

Juvenile idiopathic arthritis (JIA) is by definition an arthritis of unknown origin which is present for at least 6 weeks, starts before the age of 16, and in which other conditions have been excluded; JIA is classified into seven subtypes according to the classification criteria of the International League of Associations for Rheumatology (ILAR) (Petty et al. 2004). In any of the JIA subtypes, the temporomandibular joint (TMJ) may be affected; TMJ involvement may be present already at the time of diagnosis of JIA (Weiss et al. 2008; von Schuckmann et al. 2020), but more often it occurs with delay. The main relevance of TMJ involvement in a growing child is the increased risk for malocclusion (Huhtanen et al. 2023) and facial growth disturbance with subsequent aesthetic and functional problems.

Temporomandibular disorders (TMD) are a heterogeneous group of conditions affecting the TMJ, masticatory muscles and associated structures. According to a systematic review by Mélou et al. (2023), most published studies report a TMD prevalence of over 20% in otherwise healthy children and adolescents. The prevalence is higher in adolescents (36.9%) than in children (23.4%), and in most studies, females are more often affected than males. The review revealed several significant risk factors for TMD, including, amongst others, parafunctions (such as bruxism and nail biting), psychological factors (such as anxiety, stress, and feelings of sadness), and environmental factors (such as parental depression during the offspring´s childhood, divorced parents and single parents). The two most common diagnoses were myofascial pain and disc displacement with reduction.

The rates and types of TMJ involvement in JIA in general, and in different subtypes of JIA in particular, vary notably due to variations in study designs, included samples, and differences in clinical and imaging protocols. Moreover, there exists some heterogeneity in the terminology related to TMJ involvement, making comparison of results between studies somewhat challenging. The use of a standardised terminology for JIA-associated TMJ arthritis has therefore been recommended by the TMJaw group (Stoustrup et al. 2019), differentiating between the terms TMJ arthritis (inflammation in the joint, confirmed by magnetic resonance imaging [MRI]), TMJ involvement (abnormalities presumed to be the result of TMJ arthritis), TMJ deformity (abnormality in growth, development, or structure of the osseous and/or soft-tissue components of the TMJ), TMJ symptoms (patient- or parent-reported conditions related to TMJ arthritis or involvement), and TMJ dysfunction (physician-reported functional examination abnormalities related to TMJ arthritis or involvement).

Despite the terminological heterogeneity, the literature shows convincing evidence that JIA patients are prone to TMJ involvement, in one way or another. Studies that have included patients representing several JIA subtypes reveal rates of *“TMJ involvement”* or *“TMD”* or *“self-reported TMJ symptoms”* or *“self-reported orofacial pain”*, or *“self-reported compromised orofacial functions”*, or *“radiologically confirmed TMJ deformities or condylar lesions”* that vary between 35 and 78% (Billiau et al. 2007; Cannizzaro et al. 2011; Rahimi et al. 2018; Fischer et al. 2020; von Schuckmann et al. 2020; Collin et al. 2022). Moreover, as shown in a systematic review and meta-analysis, children and adolescents with JIA have an almost fourfold risk for TMD (RR 3.86; 95% CI 2.59–5.76) as compared to controls without JIA or any systemic disease (Ronsivalle et al. 2023).

According to Fischer et al. (2020), the highest rates of TMD in children and adolescents with JIA were observed in those with oligoarthritis (44%) and polyarthritis (24%). Oligoarthritis and polyarthritis are the two most common subtypes of JIA in Europe (Thierry et al. 2014), emphasising the importance of further analysing the characteristics of TMJ involvement in these individuals.

The main aims of the present study were to 1) describe clinical characteristics of TMJ involvement in children with oligoarthritis and seronegative polyarthritis, 2) compare clinical characteristics of TMJ involvement between children with oligoarthritis and seronegative polyarthritis, and 3) identify factors increasing the odds for different types of clinical TMJ involvements.

## Materials and methods

### Study design and sample

To address these aims, a retrospective cohort study including children, who had been diagnosed with juvenile oligoarthritis or seronegative polyarthritis at the Department of Pediatrics at Turku University Hospital, was designed and implemented. For the present analysis, children who had been diagnosed with clinical TMJ involvement during the 10-year time period between 2008 and 2017 and who had available magnetic resonance images (MRI) from around the time of TMJ involvement were included. Age under 16 years was an inclusion criterion for TMJ involvement.

### Data collection

The following data were collected from the patient files: type of JIA (i.e. oligoarthritis or seronegative polyarthritis), sex, age at the time of JIA diagnosis, age and medication when clinical TMJ involvement had been recorded, laboratory data (i.e. rheumatoid factor [RF], anticyclic citrullinated peptide [Anti-CCP], human leukocyte antigen [HLA-B27], and antinuclear antibody [ANA]), patient-reported TMJ symptoms (later referred to as symptoms, defined as any subjective symptoms related to the TMJ and masticatory muscles reported by the child), and abnormal clinical findings (later referred to as clinical findings, defined as any abnormal findings in the region of the TMJ, masticatory muscles and occlusion observed by the physician). A fellowship-trained head and neck radiologist reviewed MRIs, and any pathological findings were recorded.

### Descriptive statistics and data curation

Descriptive statistics were calculated for all variables included in the data analysis. In addition, subtypes of symptoms, clinical findings and MRI findings were identified and further classified. Symptoms were classified into the following three subclasses: pain, disturbance of mandibular movements and joint sounds. Clinical findings were classified into the following six subclasses: disturbance of mandibular movements, mandibular asymmetry, mandibular retrognathia, pain on palpation in the region of the joints or muscles, joint sounds and joint swelling. Moreover, the association between age and symptoms, clinical findings, and medication at the time of diagnosis of TMJ involvement was calculated. For this purpose, children were divided into three age groups based on when TMJ involvement had been documented for the first time; 2 to 5 years, 6 to 10 years and 11 to 15 years.

According to Tolend et al. (2021), MRI findings were grouped as follows: 1) no pathological findings, 2) inflammation (i.e. bone marrow oedema, bone marrow enhancement, joint effusion, synovial thickening, or joint enhancement), and 3) osteochondral damage (i.e. condylar flattening, erosions or disc abnormalities).

### Study variables for data analysis

The predictor variables were oligoarthritis and seronegative polyarthritis.

The primary dichotomous outcome variables were symptoms (yes/no) and clinical findings (yes/no).

The secondary dichotomous outcome variables were patient-reported pain in the region of the TMJ or masticatory muscles (yes/no) and clinically observed disturbance of mandibular movements (i.e. deviation of the mandible during mouth opening or other mandibular movements, or restriction of mandibular movements in any direction) (yes/no).

The control variables were sex, age at diagnosis of JIA, delay from the diagnosis of JIA to the date when TMJ involvement had been recorded, ANA, HLA-B27, and medication (yes/no) at the time of TMJ involvement. Based on the median age at the time of JIA diagnosis, age was further dichotomised as < 5.3 and ≥ 5.3 years. Based on the median delay from diagnosis of JIA to diagnosis of TMJ involvement, delay was dichotomised as < 33.0 and ≥ 33.0 weeks.

### Data analysis

The chi-square test was used to determine the associations between outcome variables and predictor variables and between control variables and predictor variables. When the assumptions of chi-square test were not met, the Fisher’s exact test was used. The Mann–Whitney *U *test was used for continuous variables. Multivariable logistic regression was performed to examine the associations between predictors and outcome variables, and between control variables and outcome variables. Odds ratios with 95% confidence intervals were calculated. Data analysis was performed using SPSS Statistical Package (IBM SPSS Statistics for Windows, Version 29.0.; IBM Corp., NY, USA). A *p* value less than 0.05 was set as the threshold for statistical significance.

## Results

**Fig. 1:**
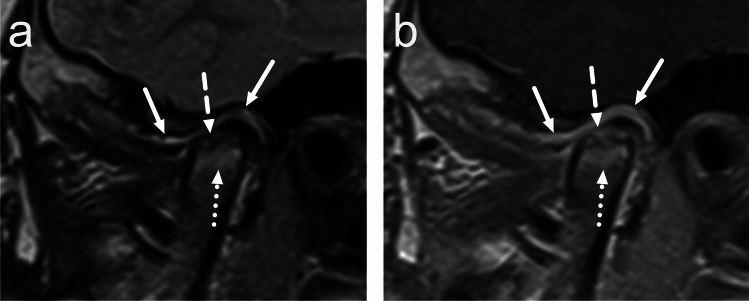
Oblique T2-weighted (**a**) and contrast-enhanced T1-weighted (**b**) MR images of the left TMJ of a 4-year-old female showing synovitis (arrows), bone marrow oedema/enhancement (dotted arrows), and condylar irregularities/erosions (dashed arrows)

In total, 62 children with TMJ involvement were identified for the present study. Descriptive statistics of the children are presented in Table 1. The majority (74.2%) were girls. The mean age at onset of JIA was 6.5 years (range 1–15, SD 3.5, median 5.3) and of TMJ involvement, 7.9 years (range 2–15, SD 3.6, median 6.7). The mean delay from diagnosis of JIA to diagnosis of TMJ involvement was 1.3 years (range less than 0–5.6 years, SD 1.6, median 33.0 weeks). In five children (8.1%), TMJ involvement was identified prior to diagnosis of JIA, leading to rheumatological investigations and subsequently to JIA diagnosis. TMJ involvement was diagnosed within a short time term after JIA diagnosis in 56.5% of the children (i.e. in 22 children 0 to 5 months and in 13 children 6 to 12 months after JIA diagnosis). With regard to laboratory parameters, ANA and HLA-B27 had been performed most frequently, with ANA having been positive in 34 children (54.8%) and HLA-B27 in 14 (22.6%). 11 children (17.7%) had no medication at the time of TMJ diagnosis. The two most common medications were NSAIDs (44 children, 71%) and synthetic disease-modifying antirheumatic drugs (sDMARDs) (42 children, 67.7%). Biological disease-modifying antirheumatic drugs (bDMARDs) were used by five children (8.1%). 42 children (67.7%) had abnormal findings on MRI (Fig. 1).

**Table 1:** Descriptive statistics of 62 children with oligoarthritis or seronegative polyarthritis and associated TMJ involvement (*N* = 62)

	Number of patients N (%)
Type of JIA	
Oligoarthritis (13 persistent, 18 extended)	31 (50.0)
Seronegative polyarthritis	31 (50.0)
Sex	
Girls	46 (74.2)
Boys	16 (25.8)
Age group at diagnosis of JIA (years)	
< 5.3	31 (50.0)
≥ 5.3	31 (50.0)
Age group at the time of TMJ involvement (years)	
2–5	27 (43.5)
6–10	20 (32.3)
11–15	15 (24.2)
< 6.7	31 (50.0)
≥ 6.7	31 (50.0)
Delay from diagnosis of JIA to diagnosis of TMJ involvement	
Before diagnosis of JIA	5 (8.1)
0–5 months after diagnosis of JIA	22 (35.5)
6–12 months after diagnosis of JIA	13 (21.0)
≥ 13 months after diagnosis of JIA	22 (35.5)
Rheumatoid factor (RF)	
Positive	0 (0.0)
Negative	30 (48.4)
Missing	32 (51.6)
Anti-cyclic citrullinated peptide antibody (Anti-CCP)	
Positive	0 (0.0)
Negative	24 (38.7)
Missing	38 (61.3)
Anti-nuclear antibody (ANA)	
Positive	34 (54.8)
Negative	28 (45.2)
Missing	0 (0.0)
Human leucocyte antigen B27 (HLA-B27)	
Positive	14 (22.6)
Negative	45 (72.6)
Missing	3 (4.8)
Medication at the time of TMJ involvement	
No medication	11 (17.7)
sDMARDs + NSAID	26 (41.9)
NSAID	9 (14.5)
sDMARDs + NSAID + CC	6 (9.7)
sDMARDs	5 (8.1)
sDMARDs + bDMARDs + NSAID	2 (3.2)
sDMARDs + bDMARDs	1 (1.6)
sDMARDs + bDMARDs + CC	1 (1.6)
sDMARDs + bDMARDs + NSAID + CC	1 (1.6)
MRI findings	
Yes	42 (67.7)
Inflammation	37 (59.7)
Osteochondral damage	39 (62.9)

*JIA* juvenile idiopathic arthritis

*TMJ* temporomandibular joint

*MRI* magnetic resonance image

*sDMARDs* synthetic disease-modifying antirheumatic drugs; methotrexate, azathioprine, hydroxychloroquine, sulfasalazine

*NSAID* non-steroidal anti-inflammatory drug

*CC* systemic corticosteroid

*bDMARDs* biological disease-modifying antirheumatic drugs; etanercept, adalimumab

The distribution of symptoms and clinical findings, and their association to oligoarthritis and seronegative polyarthritis are shown in Table 2. Overall, symptoms were far more infrequently reported (30 children, 48.4%) than clinical findings were observed (57 children, 91.9%). The most frequent subclass of symptoms was pain in the region of the TMJ or masticatory muscles (28 children, 45.2%). The most common subclass of clinical findings was disturbance of mandibular movement (46 children, 74.2%). There were no statistically significant differences in the occurrence of symptoms or clinical findings between children with oligoarthritis and seronegative polyarthritis.

**Table 2:** Association between outcome variables and primary predictor variables

	All patients (*n* = 62)	Oligoarthritis (*n* = 31)	Seronegative polyarthritis (*n* = 31)	*p* value
	*N* (%)	*N* (%)	*N* (%)	
Symptoms				0.309^a^
Yes	30 (48.4)	17 (54.8)	13 (41.9)	
Clinical findings				1.000^b^
Yes	57 (91.9)	28 (90.3)	29 (93.5)	
Subclasses of symptoms				
Pain	28 (45.2)	17 (54.8)	11 (35.5)	0.126^a^
Disturbance of mandibular movement	8 (12.9)	5 (16.1)	3 (9.7)	0.707^b^
Joint sound	3 (4.8)	3 (9.7)	0 (0.0)	0.238^b^
Subclasses of clinical findings				
Disturbance of mandibular movement	46 (74.2)	24 (77.4)	22 (71.0)	0.562^a^
Pain on palpation (joint/muscle)	18 (29.0)	11 (35.5)	7 (22.6)	0.263^a^
Mandibular asymmetry	18 (29.0)	8 (25.8)	10 (32.3)	0.576^a^
Mandibular retrognathia	10 (16.1)	6 (19.4)	4 (12.9)	0.490^a^
Joint sound	6 (9.7)	4 (12.9)	2 (6.5)	0.671^b^
Joint swelling	1 (1.6)	0 (0.0)	1 (3.2)	1.000^b^

^a^Chi-square

^b^Fisher's test

**Fig. 2:**
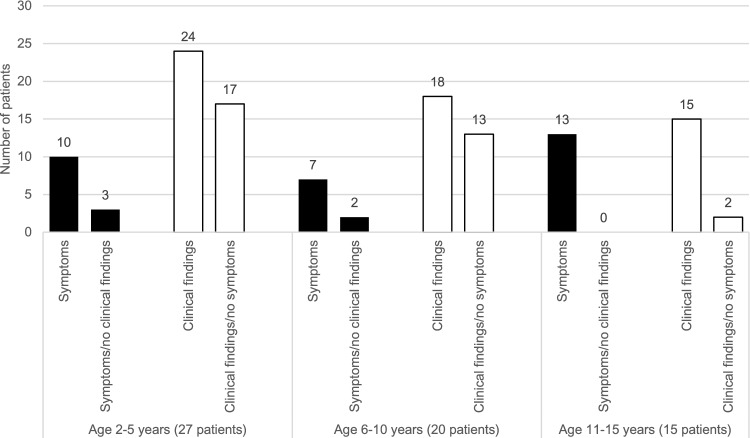
Associations between symptoms, clinical findings and age at the time of diagnosis of TMJ involvement

The association between symptoms, clinical findings and age at the time of diagnosis of TMJ involvement is illustrated in Fig. 2. The proportion of children with symptoms was far smaller in the 2- to 5-year-olds (10 of 27 children, 37.0%) and 6- to 11-year-olds (7 of 20 children, 35.0%) than in the 11- to 15-year-olds (13 of 15 children, 86.7%). Clinical findings were common in all age groups (88.9%–100%). However, out of 24 children aged 2 to 5 years with clinical findings, 17 (70.8%) did not report any symptoms, with the corresponding rate for 6- to 10-year-olds being 72.2%. The rate for 11- to 15-year-olds was 13.3%. No statistically significant differences between medication and age groups were observed (*p* = 0.223).

Table 3 presents the associations between control variables and primary predictor variables. The only control variable associated with type of JIA was sex (*p* = 0.02).

**Table 3:** Associations between control variables and primary predictor variables

	Oligoarthritis (*n* = 31)		Seronegative polyarthritis (*n* = 31)		*p* value
	*N* (%)		*N* (%)		
Sex					0.02^a^
Girls	19 (61.3)		27 (87.1)		
Boys	12 (38.7)		4 (12.9)		
Age at diagnosis of JIA (years)					
Mean		7.2		5.9	0.213^b^
Median		6.2		4.8	
Age group at diagnosis of JIA (years)					0.204^a^
< 5.3	13 (41.9)		18 (58.1)		
≥ 5.3	18 (58.1)		13 (41.9)		
Delay from diagnosis of JIA to diagnosis of TMJ involvement (weeks)					
Mean		74.4		63.7	0.757^b^
Median		38.6		25.5	
Delay from diagnosis of JIA to diagnosis of TMJ involvement (weeks)					0.204^a^
< 33.0	13 (41.9)		18 (58.1)		
≥ 33.0	18 (58.1)		13 (41.9)		
Anti-nuclear antibody (ANA)^c^					1.000^a^
Positive	17 (54.8)		17 (54.8)		
Negative	14 (45.2)		14 (45.2)		
Human leucocyte antigen B27 (HLA-B27)^d^					0.942^a^
Positive	7 (24.1)		7 (23.3)		
Negative	22 (75.9)		23 (76.7)		
Medication at the time of TMJ involvement					0.319^a^
Yes	24 (77.4)		27 (87.1)		
No	7 (22.6)		4 (12.9)		

^a^Chi-square

^b^Mann–Whitney

^c^Missing n = 0

^d^Missing n = 3

*JIA* juvenile idiopathic arthritis

*TMJ* temporomandibular joint

Multivariable logistic regression analysis for outcome variables is summarised in Table 4. ANA negative children had five times greater odds for symptoms (OR 4.88, 95% CI 1.29, 18.41, *p* = 0.019) and an almost five times greater odds for patient-reported pain (OR 4.56, 95% CI 1.21, 17.19, *p* = 0.025) as compared to children with ANA positivity. No other statistically significant differences in the odds for outcomes were observed between children with oligoarthritis and seronegative polyarthritis.

**Table 4:** Summary of multivariable logistic regression analyses for outcome variables

	Symptom present	*p* value	Patient-reported pain present	*p* value	Clinical finding present	*p* value	Clinically observed disturbance of mandibular movement present	*p* value
	OR (95% CI)		OR (95% CI)		OR (95% CI)		OR (95% CI)	
Type of JIA								
Oligoarthritis	2.29 (0.64, 8.17)	0.203	2.83 (0.78, 10.29)	0.115	1.15 (0.12, 11.00)	0.902	1.64 (0.41, 6.57)	0.483
Seronegative polyarthritis	ref		ref		ref		ref	
Sex								
Girls	3.37 (0.69, 16.42)	0.133	2.80 (0.58, 13.62)	0.202	ref		ref	
Boys	ref		ref		1.03 (0.05, 21.91)	0.983	1.18 (0.22, 6.36)	0.846
Age group at diagnosis of JIA (years)								
< 5.3	ref		ref		ref		ref	
≥ 5.3	1.28 (0.41, 3.99)	0.677	1.72 (0.54, 5.44)	0.356	3.97 (0.31, 50.79)	0.289	1.69 (0.49, 6.34)	0.440
Delay from diagnosis of JIA to diagnosis of TMJ symptoms and/or signs (weeks)								
< 33.0	1.26 (0.36, 4.50)	0.718	1.19 (0.33, 4.30)	0.792	5.68 (0.38, 85.47)	0.209	2.19 (0.53, 9.02)	0.279
≥ 33.0	ref		ref		ref		ref	
Anti-nuclear antibody (ANA)a								
Positive	ref		ref		1.77 (0.11, 28.09)	0.685	3.43 (0.79, 14.81)	0.099
Negative	4.88 (1.29, 18.41)	0.019	4.56 (1.21, 17.19)	0.025	ref		ref	
Human leucocyte antigen B27 (HLA-B27)b								
Positive	1.09 (0.29, 4.10)	0.894	1.33 (0.35, 5.13)	0.680	ref		ref	
Negative	ref		ref		7.22 (0.57, 90.85)	0.126	1.23 (0.25, 6.08)	0.798
Medication at the time of TMJ involvement								
Yes	1.73 (0.34, 8.71)	0.508	1.53 (0.29, 7.99)	0.615	NA		ref	
No	ref		ref				2.12 (0.19, 23.22)	0.537

^a^Missing n = 0

^b^Missing n = 3

*JIA* juvenile idiopathic arthritis

*TMJ* temporomandibular joint

*OR* odds ratio

*CI* confidence interval

*NA* Not applicable

*ref* reference

## Discussion

The present results revealed that abnormal TMJ findings observed by clinicians were far more frequent (57/62, 91.9%) than patient-reported symptoms (30/62, 48.4%). ANA negative children had five times greater odds for symptoms and an almost five times greater odds for patient-reported pain compared to children with ANA positivity. There were no statistically significant differences in the occurrence of symptoms or clinical findings between children with oligoarthritis and seronegative polyarthritis. Normal MRI findings were observed in only 20 children (32.3%).

An important finding in the present study was that patient-reported symptoms were far more infrequent than abnormal TMJ findings observed by the clinician; in line with the results of a study from Sweden (Cedströmer et al. 2013), which included 266 paediatric patients with JIA representing all ILAR categories. The authors observed that the occurrence of orofacial signs assessed by the physician were notably higher in all ILAR groups (57.7–92%) than the occurrence of self-reported orofacial symptoms (32–76%). In the present study, symptoms were absent far more frequently in the 2- to 10-year-olds than in the 11- to 15-year-olds, one reason being likely that the youngest children are not able to self-report. In addition, evidence suggests that parents tend to underestimate the impact of children’s oral and maxillofacial problems as their perspective is different and they might have limited knowledge of their children’s social and emotional well-being (Barbosa and Gavião 2008). However, also in the older age groups, children were observed who did not have any symptoms despite clinical findings. The reason may either be that the children did not factually have any subjective symptoms or that the symptoms were so mild that they did not acknowledge them spontaneously. The results indicate that TMJ involvement may, indeed, be present without subjective symptoms in children with JIA. Therefore, it is recommended that all physicians treating JIA patients perform a crude examination of the TMJ on a regular basis despite the absence of symptoms. If any deviations from normal are observed during the examination, the child should be referred to a more detailed examination to a dental unit having experience in the diagnosis and treatment of TMDs.

Early detection of TMJ involvement is crucial in children with JIA in order to prevent malocclusion and skeletal deformities. As reported by Ma et al. (2022), patients with JIA had a significantly higher incidence of craniofacial deformities (incidence rate 4.58) compared to matched non-JIA controls (incidence rate 2.66) on a long-term basis. In the present study, a notable number of children (18) had mandibular asymmetry at the time of diagnosis of TMJ involvement. Multidisciplinary collaboration in general and close collaboration between rheumatologists and orthodontists in particular in treatment and follow-up of children with JIA-associated TMJ involvement is essential (Rongo et al. 2023).

The present study showed that TMJ involvement frequently occurs at an early stage of JIA. 22 children (35.5%) were diagnosed with TMJ involvement in less than 6 months and 13 children (21.0%) between 6 and 12 months after a JIA diagnosis. The present results are in line with the findings of von Schuckmann et al. (2020) who observed that of 843 children with JIA and TMJ involvement, 272 (32.3%) had been diagnosed with TMJ involvement already at onset of JIA. Moreover, five children in the present study were diagnosed with TMJ involvement prior to diagnosis of JIA, leading to rheumatological investigations and subsequently to JIA diagnosis, underlining the important role of the dentist as the first detector of inflammatory joint disease.

Although several papers have been published about TMJ involvement in children with JIA, from many different aspects, we have not found studies focussing in detail on symptoms and signs of TMJ involvement in children with oligoarthritis and seronegative polyarthritis, in particular. Because JIA represents such a heterogeneous group of arthritides (Petty et al. 2004; Ravelli and Martini 2007; Long and Marston 2023), we hypothesised that clinical characteristics of TMJ involvement could differ somewhat between children with oligoarthritis and seronegative polyarthritis. However, significant differences in the occurrences of symptoms or clinical findings between groups were not observed. One reason may be the small number of children, which should be recognised as a limitation of the present study. The post hoc power analysis revealed that for a total sample size of 62, an alpha level of 0.05, and two equal-sized independent groups, the power estimated at 80% (beta = 0.20) would have been reached with proportions of 0.793 in study group and 0.42 in reference group (seronegative polyarthritis) with effect size (odds ratio) 5.29. Another limitation is that data were collected retrospectively, and no long-term follow-up parameters were collected. A Nordic study reporting temporomandibular symptoms up to 17 years after onset of JIA showed that the prevalence of TMJ involvement is, indeed, notable over the long-term (Glerup et al. 2020). Of 265 JIA patients in the study mentioned, 32.8% had at least one orofacial symptom and 51.3% had at least one sign of TMD during follow-up. However, all patients had been diagnosed during the early biological era. Further studies with larger patient materials looking into how TMJ symptoms and signs evolve over time in relation to disease activity and modern treatments are indicated.

In the present study, all children had TMJ involvement based on subjective symptoms and/or abnormal clinical findings. However, many children who had laboratory findings available did not show positive biomarkers indicating active disease. The literature reveals inconsistent results concerning the association between biomarkers and TMJ involvement in JIA patients. Abramovicz et al. (2019) investigated 60 patients aged 5 to 18 years with different types of JIA and associated TMJ involvement. Most of the patients were ANA negative, RF negative or HLA-B27 negative. Von Schuckmann et al. (2020) reported that ANA positivity was significantly associated with TMJ involvement, whereas HLA-B27-positive patients were less likely to have TMJ involvement. The results must be interpreted carefully because TMJ symptoms do not necessarily equal TMJ arthritis. Namely, in the present study, as many as 20 children (32.3%) did not have any pathological findings on MRIs whatsoever, suggesting alternative aetiological factors for joint signs and symptoms in these children.

## Conclusions

The present study focussed on clinical characteristics (i.e. patient-reported symptoms and abnormal clinical findings) of TMJ involvement in 62 children and adolescents with oligoarthritis and seronegative polyarthritis. Included were children who had been diagnosed with TMJ involvement during a ten-year time period. One of the main aims was to compare clinical characteristics between the two JIA groups, the results showing no statistically significant differences. TMJ involvement frequently occurred at an early stage of oligoarthritis and seronegative polyarthritis. TMJ involvement frequently presented without any subjective symptoms whatsoever, and one out of three children had mandibular asymmetry already at the time of diagnosis of TMJ involvement. The findings emphasise the importance of regular examinations of the TMJ in children with JIA in order to prevent facial growth disturbance and malocclusion.

## Data Availability

No datasets were generated or analysed during the current study.
